# Immune modulation of CD4^+^CD25^+^ regulatory T cells by zoledronic acid

**DOI:** 10.1186/s12865-016-0183-7

**Published:** 2016-11-25

**Authors:** Hsien Liu, Shih-Han Wang, Shin-Cheh Chen, Ching-Ying Chen, Jo-Lin Lo, Tsun-Mei Lin

**Affiliations:** 1 Department of Surgery, Chi Mei Medical Center, Liouying, Tainan, Taiwan; 2 Department of Chemical Engineering & Institute of Biotechnology and Chemical Engineering, I-Shou University, Kaohsiung, Taiwan; 3 Department of Medical Laboratory Science, I-Shou University, Kaohsiung, Taiwan; 4 Department of Surgery, Chang Gung Memorial Hospital, Taipei, Taiwan; 5 Department of Medical Research, E-DA Hospital/I-SHOU University, Kaohsiung, Taiwan; 6 Department of Internal Medicine, E-DA Hospital/I-SHOU University, Kaohsiung, Taiwan; 7 Department of Laboratory Medicine, E-DA Hospital/I-SHOU University, Kaohsiung, Taiwan

**Keywords:** Regulatory T cells, Zoledronic acid, Immunomodulation, Breast cancer

## Abstract

**Background:**

CD4^+^CD25^+^ regulatory T (Treg) cells suppress tumor immunity by inhibiting immune cells. Manipulation of Treg cells represents a new strategy for cancer treatment. Zoledronic acid (ZA), a nitrogen-containing bisphosphonate, inhibits the expression of receptor activator of nuclear factor kappa-B ligand (RANKL) on osteoblasts to inhibit osteoclastogenesis. In a mouse model of bisphosphonate-related osteonecrosis of the jaw, administration of ZA suppressed Treg-cell activity and activated inflammatory Th17 cells. However, the interaction between ZA and Treg cells remained unclear. This study investigated the immune modulation of Treg cells by ZA.

**Methods:**

Flow cytometry was used to analyze the phenotypic and immunosuppressive characteristics of Treg cells treated with ZA. Chemotactic migration was evaluated using transwell assays. Quantitative real-time PCR (qRT-PCR) was used to investigate the effect of ZA on the expression of suppressive molecules by Treg cells.

**Results:**

Proliferation of isolated Treg cells in culture was inhibited by ZA, although ZA did not induce apoptosis. qRT-PCR and flow cytometry showed that ZA significantly downregulated the expression of CCR4, CTLA4, PD-1 and RANKL on Treg cells. Chemotactic migration and immunosuppressive functions were also significantly attenuated in Treg cells pretreated with ZA, and these effects were dose-dependent. Co-culture with Treg cells significantly increased the migration rate of breast cancer cells, while pretreatment of Treg cells with ZA attenuated this effect.

**Conclusions:**

Our findings demonstrated that ZA acted as an immune modulator by significantly inhibiting the expansion, migration, immunosuppressive function and pro-metastatic ability of Treg cells. Immunomodulation of Treg cells by ZA represents a new strategy for cancer therapy.

**Electronic supplementary material:**

The online version of this article (doi:10.1186/s12865-016-0183-7) contains supplementary material, which is available to authorized users.

## Background

Regulatory T (Treg) cells comprise a subset of CD4^+^CD25^+^ T lymphocytes, and function to suppress the immune response [1, 2]. Infiltration of Treg cells into the tumor microenvironment was shown to promote tumor cell escape from immune surveillance, and contribute to tumor growth and progression, suggesting that Treg cells play an important role in the prognosis of cancer patients [3–6]. Studies investigating the mechanisms of Treg cell-mediated promotion of tumor growth showed that the differentiation, expansion, recruitment, and activation of Treg cells in tumors potently abrogated antitumor immunity and promoted local tumor growth [7–9]. It was previously reported that increased Treg-cell accumulation in breast cancer was associated with poor prognosis [4]. Using a transgenic mouse model, it was recently shown that tumor-infiltrating Treg cells are the major source of receptor activator of NF-kB ligand (RANKL), which facilitates metastasis of RANK-expressing breast cancer cells [10]. Therefore, manipulation of Treg cells to promote a more effective immune response to tumors may represent a feasible immunotherapeutic strategy for breast cancer. Furthermore, the identification of new therapeutic targets to combat tumor-induced immune suppression is dependent on elucidating the mechanisms of 1) Treg-cell trafficking and accumulation in the tumor microenvironment and 2) the interaction between cancer cells and Treg cells [11].

Zoledronic acid (ZA) is a third-generation nitrogen-containing bisphosphonate (BP) and is able to suppress osteoclastogenesis via the inhibition of RANKL expression on osteoblasts [12, 13]. Some preclinical and clinical findings suggested that ZA also has anti-tumor and anti-metastatic properties, and ZA has been reported to inhibit angiogenesis, suppress tumor cell invasion, induce tumor cell apoptosis and induce cytotoxic γδ T cells [14–19]. ZA is currently used as an adjuvant treatment for early stage breast cancers. The phase III Austrian Breast and Colorectal Cancer Study Group trial 12 (ABCSG-12) recently reported that the addition of ZA to endocrine therapy increased the duration of disease-free survival in patients with estrogen receptor-positive breast cancer. ZA was shown to reduce both local regional and distant metastases, suggesting that it might act directly on micrometastases of breast cancer cells [20]. The Zometa-Femara Adjuvant Synergy Trial (ZO-FAST), which was the largest study using ZA, also showed encouraging results when ZA was added to the adjuvant therapy of breast cancer [21]. More recently, data from the Adjuvant Zoledronic acid to reduce recurrence (AZURE) trial also suggested that ZA potentiated the activity of adjuvant endocrine therapy in postmenopausal patients [22]. Furthermore, a mouse model of ZA-related osteonecrosis of the jaw showed that ZA inhibited Treg-cell activity [23]. Although the immunomodulatory effect of ZA was proposed as being related to Treg cells, the interaction between ZA and Treg cells was unclear. The aim of this study was to investigate how ZA affected tumor immunity via Treg cells.

## Methods

### Isolation and expansion of regulatory T cells

Peripheral blood mononuclear cells (PBMCs) were isolated from 100 ml of fresh heparinized peripheral blood from healthy volunteers by Ficoll-Hypaque (GE Healthcare, Uppsala, Sweden) gradient centrifugation. The cells from the interface were washed three times in HBSS and seeded in 6-well microtiter plates in RPMI-1640 medium, and allowed to settle at 37 °C in a humidified atmosphere with 5% CO_2_. After 30 min, the non-adherent cells were collected as lymphocytes. Treg cells were immediately purified from PBMCs by immunomagnetic separation using the Dynabeads® Regulatory CD4^+^CD25^+^ T Cell Kit (Invitrogen™, Oslo, Norway), according to the manufacturer’s instructions. In brief, CD4^+^ cells were isolated by negative selection after depletion of cells expressing CD8, CD14, CD16, CD19, CD36, CDw123, CD235a and CD56. CD25^+^ cells were then selected by positive selection using magnetic beads directly conjugated to an anti-CD25 Ab. The procedure yielded a highly pure preparation (>95% purity) of regulatory CD4^+^CD25^+^ T cells, more than 80% of which expressed the intracellular transcription factor, Foxp3. The isolated Treg cells were expanded with Dynabeads® Human Treg Expander (Gibco®, Oslo, Norway) containing 100 U/ml rIL2 (Gibco®, Carlsbad, CA, USA). The study was approved by the Institutional Review Committee of E-DA Hospital and the volunteers provided written informed consent.

### Chemicals, antibodies and cell line

Zoledronic acid (ZA) was provided by Novartis Pharma AG Basel, Switzerland. Monoclonal antibodies against human CD25-PE, CD152 (CTLA-4)-PE, FOXP3-FITC, and CD279 (PD-1)-FITC were purchased from eBioscience, San Diego, CA, USA. CD194 (CCR4)-PerCP-CyTM 5.5, and CD69-FITC were purchased from BD Biosciences, San Jose, CA, USA. The MDA-MB-231 breast cancer cell line was purchased from the American Type Culture Collection (Food Industry Research and Development Institute, Taiwan) and was cultured with DMEM medium (Gibco®, Grand Island, NY, USA), containing 10% fetal bovine serum (FBS: Biological industries, Kibbutz Beit HaEmek, Israel). Conditioned media (CM) harvested from MDA-MB-231 cells consisted of DMEM supplemented with 2% FBS in which cells were cultured for 48 h. CM was collected, sterile filtered and stored in aliquots at −80 °C.

### Proliferation assay

Expanded Tregs and lymphocytes were labelled with 2 μM carboxyflourescein diacetate succinimidyl ester (CFSE) (BD Biosciences, San Jose, CA) at 37 °C for 15 min. The cells were seeded in 12-well plates at a density of 2.5 × 10^6^ cells/well, cultured with culture medium in 0, 10 and 100 μM ZA, and stimulated with CD3/CD28 micro beads. The culture media was changed every 3 days and measured. They were analyzed using the Cell Quest Pro software (BD Biosciences, San Jose, CA) or WinMDI software.

### Treg cells apoptosis assays

Apoptosis of Treg cells was analyzed by seeding Treg cells at a density of 5 × 10^5^cells/well, and starving them overnight in serum free DMEM. The cells were then treated with ZA (0 or 100 μM) for 24 h in DMEM supplemented with 2% FBS. Cells were harvested and incubated with FITC-labeled Annexin V and Propidium Iodide Staining Solution (eBioscience, San Diego, CA, USA). The percentages of apoptotic and necrotic cells were determined by flow cytometry (FACScalibur; BD Biosciences, San Jose, CA, USA). Five parallel samples were measured and 10,000 events were analyzed using Cell Quest Pro software (BD Biosciences, San Jose, CA, USA) or WinMDI software (San Diego, CA, USA). In addition, Treg cells were centrifuged onto microscope slides using a Cytospin2 centrifuge (Shandon Inc., Pittsburgh, PA, USA); the slides were stained with Wright-Giemsa solution and observed by light microscopy (Olympus, Tokyo, Japan).

### Treg cells migration assays

Migration assays were performed in 24-well transwell chambers (Corning, New York, NY, USA) using 8-μm pore polycarbonate filters. Treg cells pre-treated for 4 h with different concentrations of ZA (0, 50 and 100 μM) were added to the inner chamber in serum-free RPMI medium in a final volume of 100 μL. The chemoattractants, including 2% FBS-containing DMEM or CM from MDA-MB-231 cells, were placed in the bottom chamber of the transwells in a total volume of 650 μL. Transwell chambers were incubated at 37 °C for 2 h, and the number of cells in the lower chamber was counted. The chemotaxis index was calculated by dividing the numbers of cells migrated in response to CM of MDA-MB-231 cells or after pretreatment with ZA by the numbers of cells migrated in response to medium only.

### Wound healing migration assay

For wound healing migration assays, MDA-MB-231 cells were cultured in 6-cm dishes at a density of 1 × 10^6^ cells/dish until confluent (0 h). A straight scratch was made in the center of the plate using a 200 μL pipette-tip. The MDA-MB-231 cells were then incubated for 12 h with Treg cells, which had been pre-treated with ZA (0, 25, 50 or 100 μM) for 24 h. Wound healing was evaluated by microscopy. The average cell migration distances were measured using an inverted phase-contrast microscope (Zeiss, Primovert, Germany).

### Immunosuppressive function assays

Treg cells were cultured in medium or CM of MDA-MB-231 cells for 72 h and treated with different concentrations of ZA for 24 h. Their immunosuppressive activity was determined with a Human Regulatory T Cell Function Kit (BD Biosciences, San Jose, CA, USA). In brief, the harvested Treg cells (2x10^5^) were co-cultured in 24-well plates with responding effector cells (PBMC 4x10^5^) in the presence of T-cell specific stimulated CD3/CD28 beads. FITC-labeled CD69 was present for the entire duration of stimulation in order to analyze the transiently expressed CD69 on the surface of the effector cells. After 7 h of activation, the percentage of CD69-positive effector T cells was determined by flow cytometry [24]. The percent suppression was calculated by the following formulas: 100–[(% CD69-positive in presence of Tregs/% CD69-positive in absence of Tregs) × 100].

### Quantitative real-time polymerase chain reaction assay

Treg cells were treated with various concentration of ZA for 6 h, and total RNA was extracted using the total RNA mini kit (Viogene, Sunnyvale, CA, USA) according to the manufacturer’s instructions. Samples of total RNA (1–2.5 μg) were reverse-transcribed using iScriptTM cDNA Synthesis Kit (Bio-Rad, Foster City, CA, USA) according to the manufacturer’s instructions. Real-time PCR was carried out (iTaqTM Universal SYBR@ Green Supermix; Bio-Rad, Foster City, CA, USA) with primers specific for the human gene (Table 1). Fluorescence RT-PCR analysis was performed using ABI PRISM 7700 Sequence Detection System (Applied Biosystems, Warrington, WA, USA). PCR amplification was performed with 1 cycle of 50 °C for 2 min, 1 cycle of 95 °C for 15 min, and 40 cycles of 95 °C for 15 s followed by 58 °C for 1 min. Analysis of relative gene expression was performed using the 2-ΔΔCT method [25]. The CT is the threshold cycle number that is the minimal cycle for sample detection. The arithmetic formula for the ΔCT method is the difference in CT between the detected gene and the GAPDH housekeeping gene.

**Table 1:** Primer sequences used for quantitative RT-PCR for mRNA expression

Target gene	Oligonucleotide sequences (5′-3′)	
	Forward	Reverse
Foxp3	TGA CCA AGG CTT CAT CTG TG	GAG GAA CTC TGG GAA TGT GC
CCR4	CTT CAG TCA CCT GGC TGT CA	CTC AGC TGA ACC TGG CTA CC
TGF-β	GGA AAC CCA CAA CGA AAT C	GCT CTG ATG TGT TGA AGA AC
RANKL	ACC AGC ATC AAA ATC CCA AG	CCC CAA AGT ATG TTG CAT CC
CTLA-4	CTC AGC TGA ACC TGG CTA CC	CCA CTT GCA GAC ACC ATT TG
PD-1	ATC AAA GAG AGC CTG CGG G	GGT GGG CTG TGG GCA CT
GAPDH	TGA ACG GGA AGC TCA CTG G	TCC ACC ACC CTG TTG CTG TA

### Statistical analysis

Results represent at least 3 independent experiments. Results are presented as mean ± SD. The statistical analysis was performed using Prism version 5.00 software (GraphPad Software, USA). All comparisons were made between two groups with the two-tailed Student’s *t*-test, among more than three groups with one-way ANOVA. *P* values of <0.05 were considered statistically significant.

## Results

### ZA inhibits proliferation of Treg cells

**Fig. 1:**
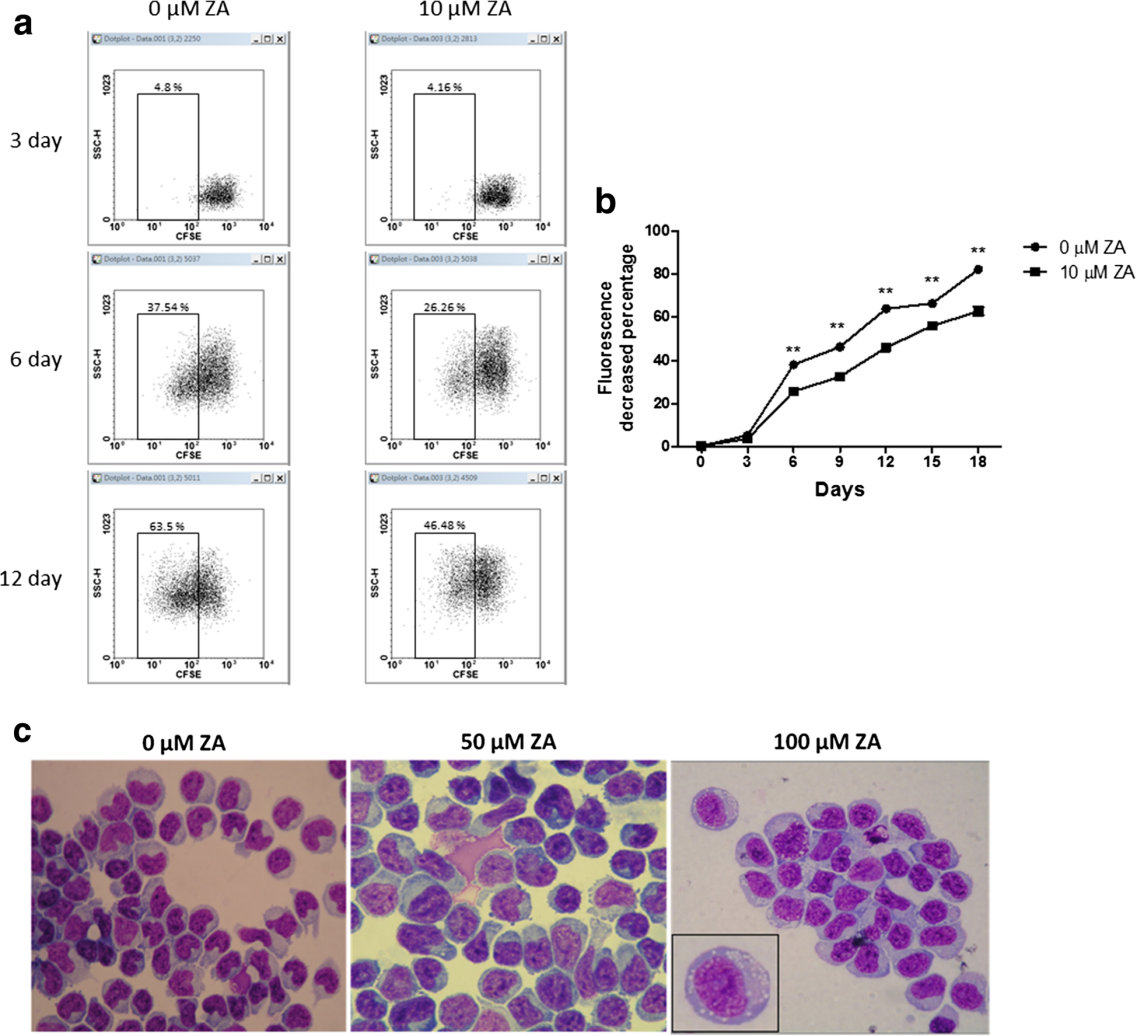
ZA inhibits Treg cells proliferation and induces cell injury. **a** Expanded Treg cells were labeled with CFSE and cultured in Treg cell medium with or without 10 μM ZA. **b** Treg cell proliferation curves were measured based on the percentage of cells with decreased fluorescence as compared to non-proliferating cells (0.38% at day 1). Data represent the mean values ± SEM and results from three independent experiments are shown. Statistical significance (*P* < 0.01) is denoted by **. **c** The morphology of Treg cells was analyzed by microscopy in 100× oil immersion after ZA treatment for 24 h

Expended Treg cells and freshly isolated lymphocytes were treated with 10 μM ZA in order to evaluate the effect of ZA on Treg-cell proliferation. CD4+ lymphocytes proliferation demonstrated no difference in the presence of 10 μM ZA (Additional file 1: Figure S1). In contrast, Treg-cell proliferation was significantly suppressed in the presence of 10 μM ZA (Fig. 1a). Inhibition of proliferation was observed as early as 6 days after ZA treatment Treatment with 10 μM ZA for 12 days inhibited proliferation by more than 28% (Fig. 1b). In addition, Treg cells treated with ZA for 24 h exhibited abundant cytoplasmic vacuoles, suggesting survival stress and early cell injury (Fig. 1c). However, annexin V and PI staining showed no evidence of apoptosis even in cells treated with 100 μM ZA for 24 h (Additional file 2: Figure S2).

### ZA inhibits chemotactic migration of Treg cells

**Fig. 2:**
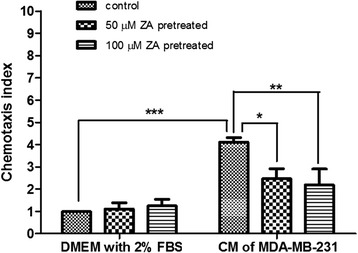
ZA inhibits Treg cells chemotactic migration. Treg cells (5 × 10 ^4^) were pretreated with 0, 50 or 100 μM ZA for 4 h, and placed in the upper chambers. Migration of Treg cells into the lower chambers containing DMEM with 2% FBS or CM from MDA-MB-231 cells after 2 h was analyzed. The chemotaxis index shown compares migration with the response of control cells to DMEM with 2% FBS. Values are means ± SEM of results from three independent experiments in duplicate. *P < 0.05, ** *p* < 0.01, ***p < 0.001

Transwell assays were used to evaluate the effect of ZA on the chemotactic migration of Treg cells in response to DMEM supplemented with 2% FBS or CM from MDA-MB-231 cells. We found that MDA-MB-231 cell CM had a greater (4.12 ± 0.19 folds) increase in Treg-cell chemotaxis compared with DMEM with 2% FBS (*p* < 0.001). ZA pretreatment significantly inhibited migration of Treg cells in response to CM from MDA-MB-231 cells. However, the migration of ZA-pretreated Treg cells was not significantly affected in the presence of DMEM containing 2% FBS (Fig. 2).

### ZA alters the phenotypic expression of Treg cells

**Fig. 3:**
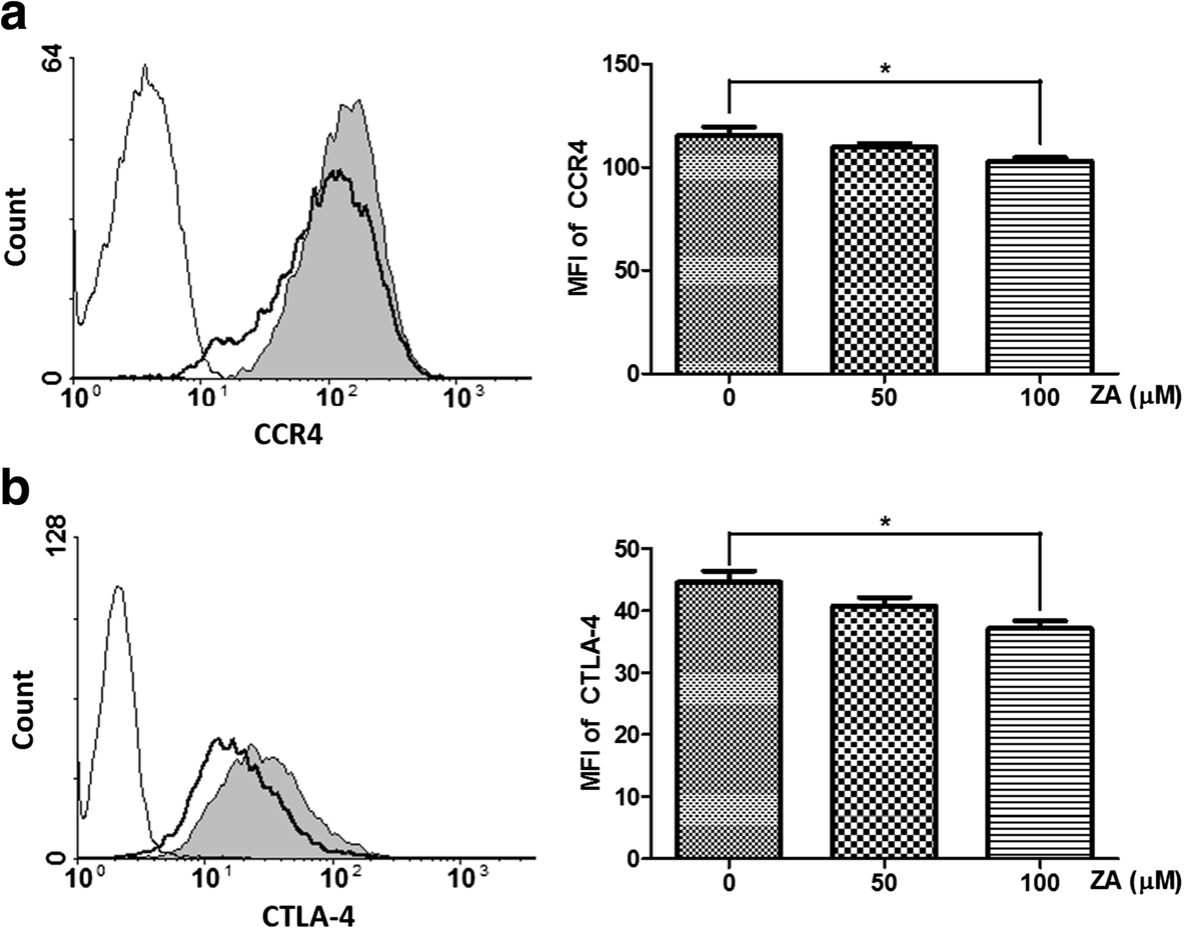
ZA influences the expression of markers associated with Treg cells suppressive function. Representative flow cytometry results of (**a**) CCR4 and (**b**) CTLA4 on Treg cells treated with 100 μM ZA (*dotted line*) and untreated Treg cells (shaded histogram). The negative control (unstained Treg cells) is shown as an open histogram. Median fluorescence intensity (MFI) is plotted against the ZA concentration (μM); the results from four independent experiments are shown. Significant difference in MFI determined using the one-way ANOZA;*p < 0.05

**Fig. 4:**
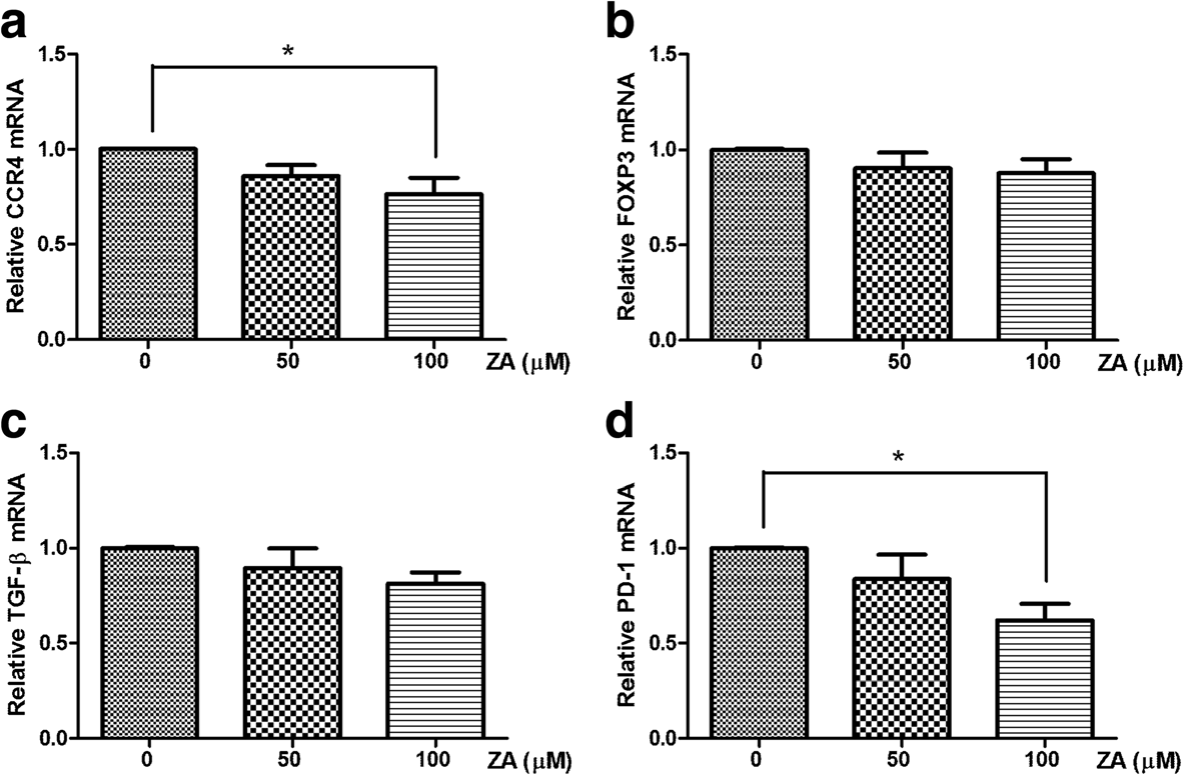
Influence of ZA on mRNA expression by Treg cells. mRNA levels were evaluated by qRT-PCR in Treg cells treated with 0, 50, 100 μM ZA for 6 h. The gene expression values were normalized to GAPDH expression. The relative mRNA expression of (**a**) CCR4, (**b**) Foxp3, (**c**) TGFβ and (**d**) PD-1 was calculated from Treg cells treated with ZA compared with untreated Treg cells. Values shown are means ± SEM of results from four independent experiments performed in duplicate. *p < 0.05

The affinity between chemokine (C-C motif) ligand 2 (CCL2) released by tumor cells and chemokine (C-C motif) receptor 4 (CCR4) expressed on Treg cells has been shown to play a major role in the recruitment of Treg cells to tumor sites [26, 27]. Cytotoxic T-lymphocyte antigen 4 (CTLA4), a surface protein receptor associated with the transmission of an inhibitory signal to T cells, is expressed on functional Treg cells [28, 29]. Thus, these phenotypic characteristics of Treg cells were analyzed by flow cytometry after treatment with ZA. We found a significant decrease in the expression of CCR4 and CTLA4 on Treg cells after treatment with 100 μM ZA (Fig. 3). In addition, 100 μM ZA treatment significantly decreased the mRNA expression of CCR4, but not Foxp3 (Fig. 4a, b) as determined by qRT-PCR. Transforming growth factor beta (TGF-β) and programmed cell death 1 (PD-1) are negative regulators of T cell immune responses required for maintaining peripheral tolerance by Treg cells [30–32]. We showed that Treg cells treated with ZA exhibited a significant decrease in the expression of PD-1 but not TGF-β (Fig. 4c, d). Therefore, ZA effectively altered the phenotype and function of Treg cells, in vitro.

### ZA inhibits the ability of Treg cells to sustain breast cancer cell migration

**Fig. 5:**
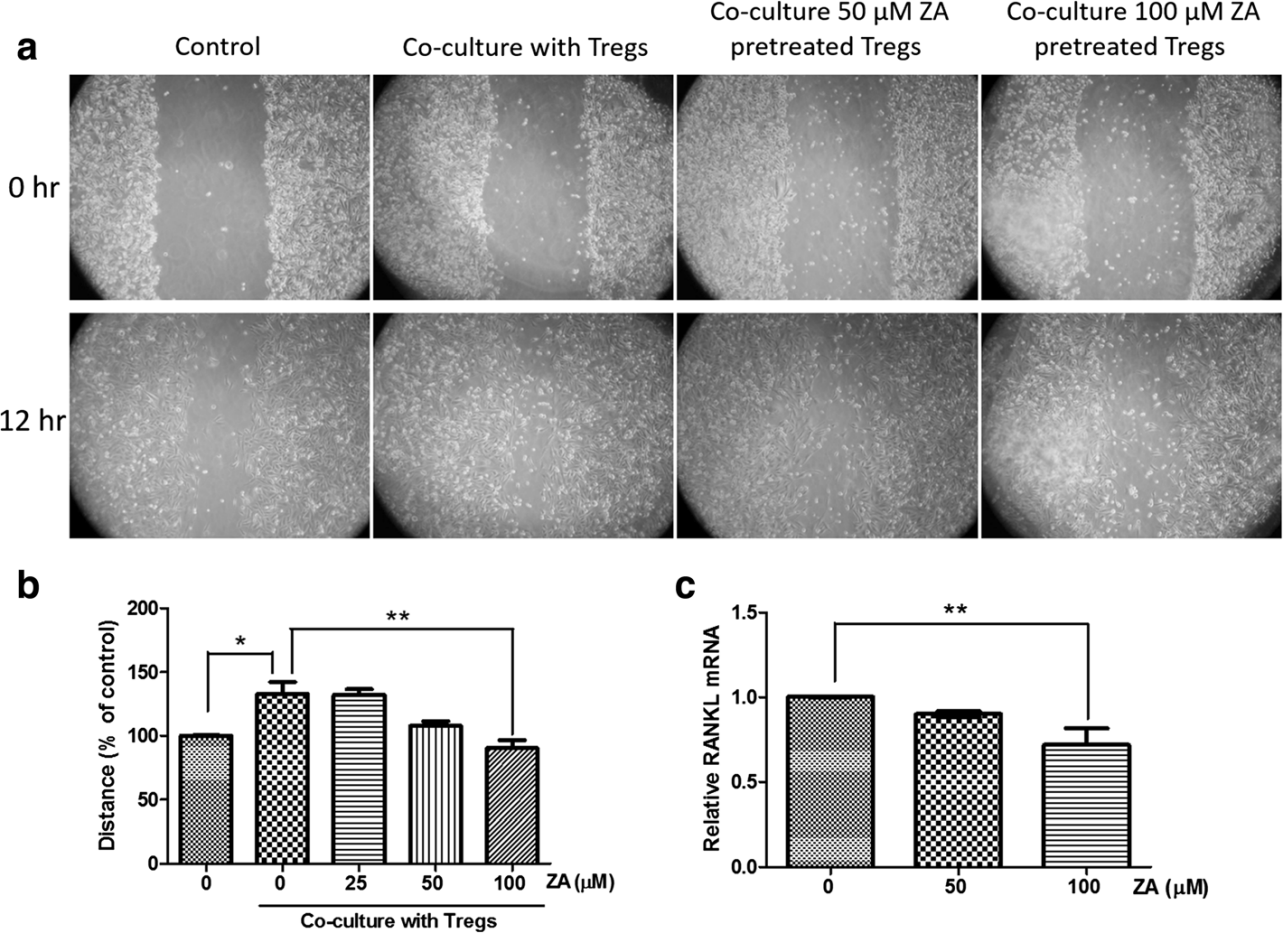
Effects of ZA on the ability of Treg cells to enhance MDA-MB-231 cell migration. **a** Representative pictures of migration (wound closing) of MDA-MB-231 cells grown as a mono-culture or co-culture with Treg cells at 0 and 12 h after wounding (100× magnification). **b** Quantification of the migration distance as a percentage of the control (MDA-MB-231 cells only). **c** The relative RANKL mRNA expression was evaluated by qRT-PCR from Treg cells treated with 0, 50, or 100 μM ZA for 6 h. The results from three independent experiments are shown. *P < 0.05, **p < 0.01

We investigated whether the ability of Treg cells to enhance the migration of breast cancer cells was modulated by ZA treatment. Wound healing assays performed with MDA-MB 231 cells showed that co-culture with Treg cells significantly increased the migration rate of breast cancer cells, compared with MDA-MB 231 cells alone (132.8 ± 9.5%, *P* < 0.05; Fig. 5a, b). However, when MDA-MB-231 cells were co-cultured with Treg cells pretreated with ZA, there was a significant and dose-dependent decrease in their migration compared to tumor cells co-cultured with untreated Treg cells (Fig. 5a, b). In particular, Treg cells pretreated with 100 μM ZA showed pronounced inhibition of MDA-MB-231 cell migration (Fig. 5b; *p* < 0.05).

The metastatic spread of carcinoma cells is related to the production of RANKL by Treg cells. ZA treatment was previously shown to downregulate the expression of RANKL by osteoblasts [12, 13]. Using qRT-PCR, we showed that ZA treatment downregulated the expression of RANKL by Treg cells in a dose-dependent manner (Fig. 5c). These results implied that ZA-mediated downregulation of RANKL by Treg cells might attenuate the ability of Treg cells to promote the migration of MDA-MB-231 cells.

### ZA inhibits immunosuppressive function of Treg cells

**Fig. 6:**
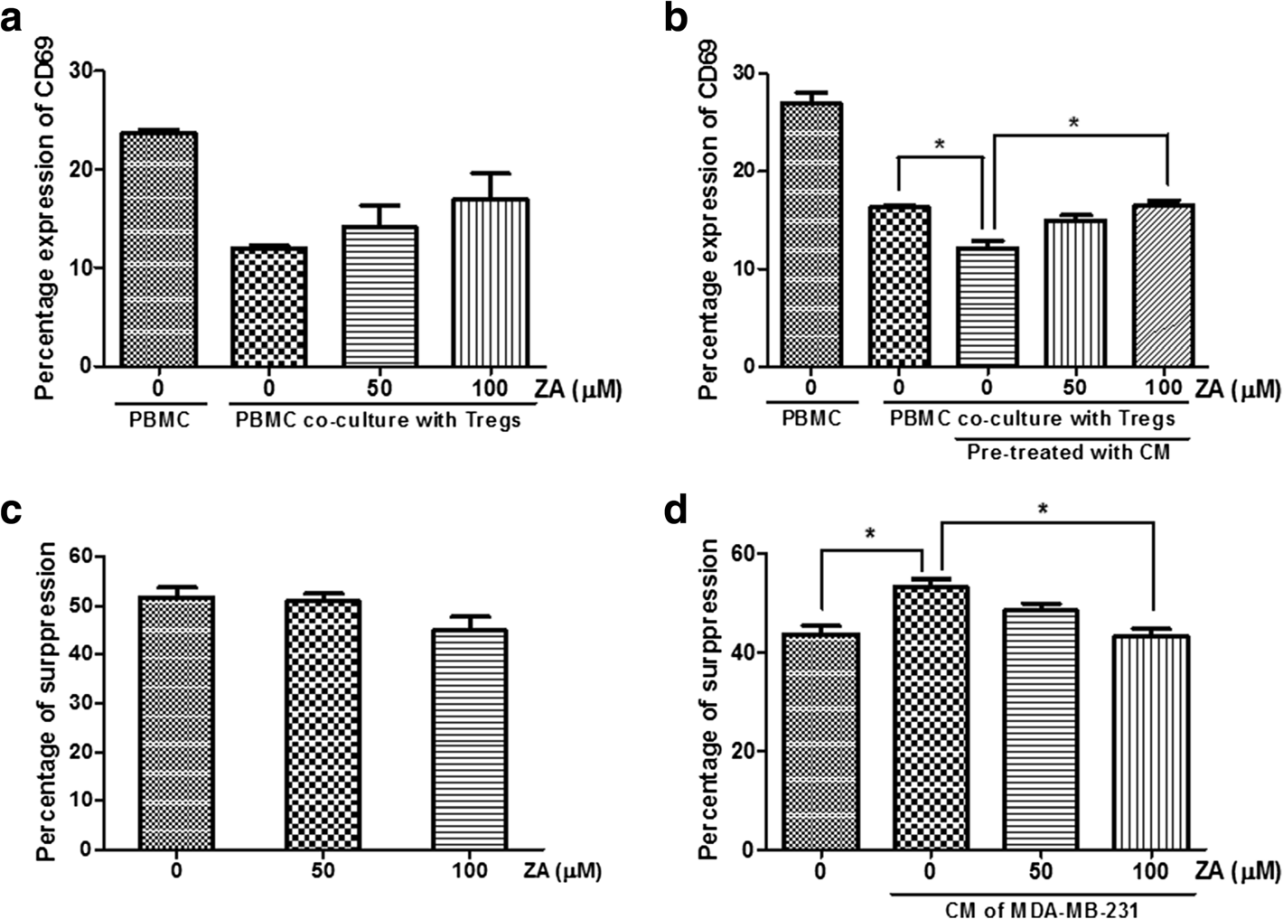
ZA inhibits the immunosuppressive function of Treg cells. **a** Treg cells were treated with 0, 50, or 100 μM ZA for 24 h, and co-cultured with autologous anti-CD3/CD28-stimulated PBMCs for 7 h. The percentage of CD69 expression on effector T cells was analyzed by flow cytometry. **b** Treg cells were pretreated with CM from MDA-MB-231 cells for 72 h, followed by 0, 50, 100 μM ZA treatment for 24 h. The Tregs were then co-cultured with autologous anti-CD3/CD28-stimulated PBMCs for 7 h. The percentage of CD69 expression on effector T cells was analyzed by flow cytometry. **c** and **d** The percent suppression of the Tregs on effector T-cell without (**c**) and with (**d**) pretreated by CM from MDA-MB-231 cells after ZA treatment. The percent suppression was calculated by the following formula: 100–[(% CD69-positive in presence of Tregs/% CD69-positive in absence of Tregs) × 100]. Values shown are means ± SEM of results from four independent experiments performed in duplicate. *P < 0.05

CD69 is an early activation marker on effector T cells, thus we investigated whether isolated Treg cells could suppress T cell activation after 7 h in culture. PBMCs were cultured either alone or together with autologous isolated Treg cells at a 2:1 ratio in the presence of anti-CD3/CD28–coated microbeads and CD69 expression was measured by flow cytometry. The results show that pretreatment of Treg cells with 100 μM ZA inhibited their immunosuppressive function, as evidenced by increase expression of CD69 (16.9 ± 3.8% vs. 12.0 ± 0.5%) (Fig. 6a), the percentage of Treg suppression was significantly decreased (51.8 ± 4.0% vs. 44.9 ± 5.6%) (Fig. 6c). Interestingly, pretreatment of Treg cells with CM from MDA-MB-231 cells significantly inhibited CD69 expression on effector T cells (16.3 ± 0.4% vs. 12.1 ± 1.3%; *p* < 0.05), but the suppressed effect was reversed and increased by ZA treatment in a dose-dependent manner (Fig. 6b). The percent suppression of CM pretreated Treg cells were significantly attenuated by 100 μM ZA treatment (Fig. 6d).

## Discussion

Patients with early-stage breast cancer and multiple myeloma have been shown to benefit from the addition of ZA to adjuvant endocrine therapy, or to standard chemotherapy, respectively [20, 33]. Previous reports have also demonstrated the efficacy of ZA therapy in postmenopausal women, although the underlying mechanism is not fully understood [34, 35]. ZA treatment for osteoporosis is frequently associated with the onset of an acute phase reaction to the activation of γδ T cells [36]. In contrast, human γδ T cells have been shown to display potent cytotoxicity against various tumor cells after pretreatment with ZA [19]. There is increasing evidence to suggest that Treg cell-mediated immunosuppression plays an important role in the progression and metastasis of breast cancer, and immune escape by tumor cells is the major challenge to current immunotherapeutic strategies [37]. These studies suggested that there is an urgent need to increase the efficacy of immunotherapy by exploring therapeutic interventions that inhibit the function of Treg cells in tumor-bearing hosts [11]. The effect of ZA on the immune modulatory function of Treg cells was previously not well understood. Previous studies using ZA treatment on other cells (oral keratinocytes and osteosarcoma cells) demonstrated cell cycle arrest in S phase through various mechanisms. [38, 39] In this study, we showed that ZA inhibited the proliferation and chemotactic migration of Treg cells, thereby improving the antitumor immune response. Recently, Ghigo et al. found a similar effect of ZA, which down-regulated indoleamine 1,2 dioxygenase and enabled the proliferation of T cells and inhibited the expansion of Treg cells [40].

We evaluated the effects of ZA on Treg cells suppressive function and gene expression, in vitro. Treg cells are recruited to the stroma of developing breast tumors and play a fundamental role in the pathogenesis of metastasis [10]. In this study, we demonstrated for the first time that the migration of Treg cells towards cancer cells was significantly inhibited by ZA. ZA also inhibited the expansion and immunosuppressive function of Treg cells in vitro. Our data suggested that the ZA-mediated attenuation of immune evasion and tumor progression was effected via inhibition of Treg cell recruitment by primary tumors, and a decrease in the number of tumor-infiltrating Treg cells. Studies on bisphosphonate-related osteonecrosis of the jaw (BRONJ) has shown that administration of ZA and dexamethasone caused BRONJ-like symptoms in mice, and this was attributed, in part, to suppression of Treg cells and activation of the inflammatory T-helper-producing interleukin 17 cells (Th17). They demonstrated systemic infusion with mesenchymal stem cells or thymus-derived Tregs could prevent and cure BRONJ-like disease via inhibition of Th17 and increase in Tregs. In addition, Tregs have been shown in vitro and in vivo to suppress Th17 cells; the BRONJ-like mice model showed that treatment with Zol/Dex suppressed both Tregs and the Treg/Th17 ratio in peripheral blood [23]. ZA not only inhibited Treg-cell function, but also enhanced cellular immunity via expansion of cytotoxic γδ T cells and MHC-restricted αβ CD8^+^ T cells [41]. These data, along with ours, suggested that ZA induced immune modulation to suppress Treg-cell functions. To the best of our knowledge, we are the first to demonstrate that treatment with ZA blocked the RANK/RANKL pathway to significantly inhibit cancer cell migration induced by Treg cells. Our data were consistent with previous studies, which suggested that strategies to deplete Treg cells in breast cancer patients [42, 43] may also be beneficial by reducing local immunosuppression and removing a primary source of RANKL required for tumor metastasis.

The concentrations of ZA used in our experiments (25–100 μM) were much higher than the plasma levels that are observed following intravenous administration of the drug (about 1–2 μM) [44]. Since ZA mainly accumulates in bone tissue and is retained for long periods of time, it is possible that ZA can reach high concentrations within the bone marrow microenvironment where large numbers of functional Treg cells accumulate [45]. In fact, bone marrow is a reservoir for Treg cells that traffic through CXCL12/CXCR4 signals [46]. We demonstrated that there may be two different mechanisms underlying the anti-tumor effect of ZA in breast cancer. Inhibition of Treg-cell migration by ZA could significantly impair the recruitment of Treg cells by breast cancer cells, resulting in reduced progression of micrometastatic foci in soft tissues. Alternatively, ZA could act on breast cancer micrometastases within the bone marrow. Although it is possible that ZA in this latter model might directly inhibit breast cancer cell growth, it is more likely that ZA-mediated inhibition of factors produced by Treg cells, which promote cancer cell migration, significantly hampers the ability of tumor cells to form distant metastases [10].

The level of Foxp3 protein expression in Treg cells is critical for immune suppression, and knockdown of Foxp3 was previously shown to result in impaired immune suppression [47]. CTLA4 and PD-1 expression by Treg cells is constitutive and critical to their suppressive function [29, 32]. Additionally, the RANK-RANKL signaling pathway is critically involved in regulating the function of Treg cells in a mouse model of colitis [48]. These studies were all consistent with our data showing that downregulation of CTLA4 and RANKL resulted in inhibition of Treg cells’ immunosuppressive function.

## Conclusions

Patients with early-stage breast cancer and multiple myeloma have been shown to benefit from the addition of ZA to adjuvant endocrine therapy, or to standard chemotherapy. In this study, we demonstrated that ZA influenced the suppressive activity of Treg cells and significantly affected the ability of Treg cells to secrete RANKL, which is known to promote breast cancer cell migration. ZA acted as an immune modulator by significantly inhibiting the expansion, migration, immunosuppressive function and pro-metastatic ability of Treg cells. Our data suggested that immunomodulation of Treg cells by ZA is a novel strategy for cancer therapy.

## Additional files

Additional file 1: Figure S1. The effect of ZA on CD4+ lymphocyte proliferation. (A) Isolated lymphocytes were labeled with CFSE, cultured in medium with or without 10 μM ZA and sensitized with anti-CD3 and anti-CD28 antibodies. (B) CD4+ lymphocyte proliferation curves were measured based on the percentage of cells with decreased fluorescence as compared to non-proliferating cells (0.4% at day 1). Data represent the mean values ± SEM and results from three independent experiments are shown. (TIF 134 kb)
Additional file 2: Figure S2. The effect of ZA on Treg cells apoptosis. (A) Treg cells were treated with and without ZA (10, 50 and 100 μM) for 24 h. Apoptosis was measured by Annexin V‑FITC/PI staining and flow cytometry. (B) Quantitative analysis of Treg cells by flow cytometry revealed no effect of ZA on Treg cell apoptosis. (TIF 131 kb)

## Abbreviations

BP: Bisphosphonate
CM: Conditioned media harvested from MDA-MB-231 cells consisted of DMEM supplemented with 2% FBS in which cells were cultured for 48 h
CTLA4: Cytotoxic T-lymphocyte antigen 4
PBMCs: Peripheral blood mononuclear cells
PD-1: Programmed cell death 1
RANK: A receptor activator of NF-kB
TGF-β: Transforming growth factor beta
Tregs: Regulatory T cells
ZA: Zoledronic acid
