# Supplementary figures and images for: Immune modulation of CD4+CD25+ regulatory T cells by zoledronic acid
Source: BMC Immunol. 2016 Nov 25;17:45. doi: 10.1186/s12865-016-0183-7 (PMC5124310; doi:10.1186/s12865-016-0183-7)

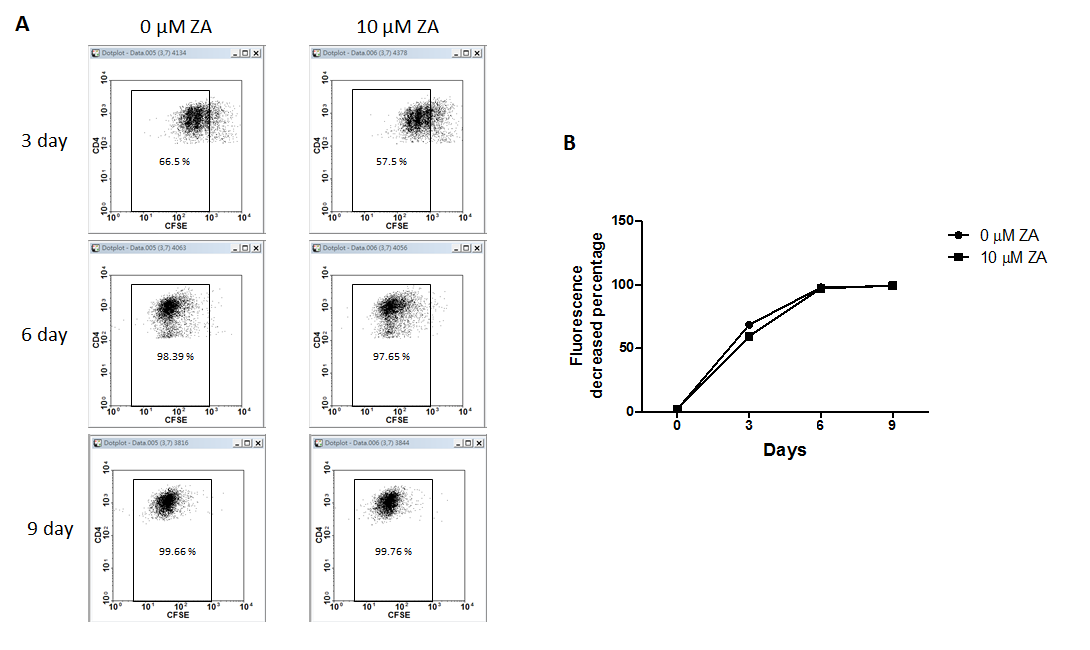

Supplement: Additional file 1: Figure S1. — The effect of ZA on CD4+ lymphocyte proliferation. (A) Isolated lymphocytes were labeled with CFSE, cultured in medium with or without 10 μM ZA and sensitized with anti-CD3 and anti-CD28 antibodies. (B) CD4+ lymphocyte proliferation curves were measured based on the percentage of cells with decreased fluorescence as compared to non-proliferating cells (0.4% at day 1). Data represent the mean values ± SEM and results from three independent experiments are shown. (TIF 134 kb) [file 12865_2016_183_MOESM1_ESM.tif]

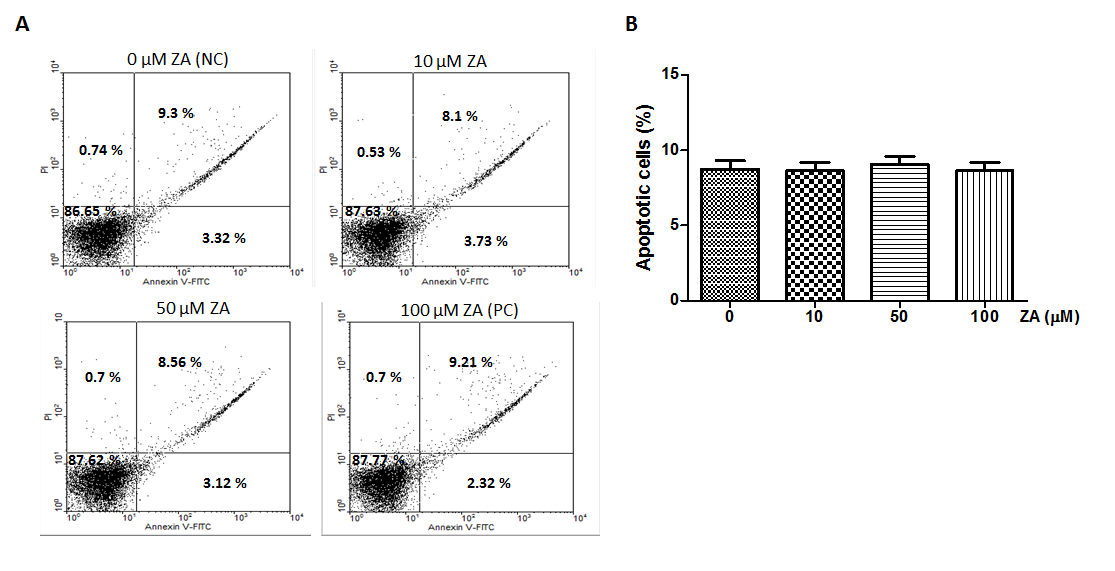

Supplement: Additional file 2: Figure S2. — The effect of ZA on Treg cells apoptosis. (A) Treg cells were treated with and without ZA (10, 50 and 100 μM) for 24 h. Apoptosis was measured by Annexin V‑FITC/PI staining and flow cytometry. (B) Quantitative analysis of Treg cells by flow cytometry revealed no effect of ZA on Treg cell apoptosis. (TIF 131 kb) [file 12865_2016_183_MOESM2_ESM.tif]
